# 3D-Printed Plantar Orthoses and the Conditional Viability of Recycled PLA

**DOI:** 10.3390/biomimetics11060414

**Published:** 2026-06-11

**Authors:** Elena Arce, Silvia Losada-Pérez, Rosa Devesa-Rey, Miguel Ángel Álvarez-Feijoo, Pablo Agregán, Raquel Leirós-Rodríguez

**Affiliations:** 1 Department of Industrial Engineering, Ferrol Polytechnic School of Engineering, University of A Coruña, 15403 Ferrol, Spain; pablo.agregan@udc.es; 2 Department of Design, School of Industrial Engineering, University of Vigo, 36310 Vigo, Spain; silviamaria.losada@uvigo.gal (S.L.-P.); alvarezfeijoo@uvigo.gal (M.Á.Á.-F.); 3 University Defense Center at Spanish Naval Academy, University of Vigo, 36920 Marin, Spain; 4 Department of Nursing and Physical Therapy, Faculty of Health Sciences, University of León, 24401 Ponferrada, Spain; rleir@unileon.es

**Keywords:** plantar orthoses, 3D printing, additive manufacturing, recycled PLA, circular economy, customized insoles

## Abstract

Plantar orthoses play an important role in podiatric care, as they help to redistribute plantar loads, improve foot function, and support the treatment of various conditions, including diabetic foot disease. In this context, additive manufacturing has substantially expanded the capacity to produce customized orthoses through digital geometry acquisition, computational design, and controlled fabrication. From a biomimetic and bionic perspective, 3D-printed plantar orthoses can be understood as engineered interfaces that reproduce, support, or modulate key biomechanical functions of the human foot, including load redistribution, shock attenuation, adaptive stiffness, and gait stabilization. Additive manufacturing enables these biological and biomechanical principles to be translated into patient-specific devices through controlled geometry, graded structures, and material selection. Moreover, from a sustainability perspective, recycled polylactic acid (rPLA) has emerged as a material of potential interest for this type of application, not only because of its compatibility with 3D-printing processes but also because it offers the possibility of reusing polymer waste and reducing the consumption of virgin raw materials in devices whose service life may be limited. This review examines the conditional viability of recycled PLA for 3D-printed plantar orthoses by integrating direct clinical evidence on orthotic function with indirect technical evidence from material-level and process-level studies. The reviewed literature indicates that recycled PLA may offer environmental and economic benefits; however, repeated thermomechanical reprocessing may alter viscosity, dimensional consistency, crystallinity, interlayer adhesion, and mechanical reliability. Recent orthosis-focused studies show that extrusion-based technologies can be applied to customized insoles, lattice or internally reinforced structures, multimaterial systems, and emerging smart concepts; however, most of these developments still rely on virgin or ad hoc-designed materials rather than recycled feedstocks. Overall, the available evidence suggests that recycled PLA should not yet be regarded as a direct substitute for virgin PLA in plantar orthoses. At present, the evidence supporting the use of recycled PLA in plantar orthoses is predominantly indirect and technical rather than directly clinical. Its use appears technically promising, but its viability remains conditional and depends on feedstock traceability, control of the manufacturing process, the suitability of material properties for device function, and validation of the orthosis under clinical conditions.

## 1. Introduction

Many patients develop foot, knee, or spinal disorders associated with mechanical abnormalities that can be managed using plantar orthoses. These devices are widely used in conservative podiatric care because they help redistribute plantar pressure, optimize load transfer during gait, protect tissues and improve functional performance. Their role is especially relevant in diabetes-related foot disease, where neuropathy, deformity, and repetitive mechanical stress increase the risk of ulceration and recurrence [1,2,3,4].

The manufacture of custom foot orthoses has traditionally relied on physical impressions of the foot, obtained using phenolic foam or plaster casts, followed by thermoforming or manual finishing [5]. More recently, digital workflows have incorporated three-dimensional scanning, baropodometric analysis, computer-aided design, and computer numerical control machining. These approaches improve reproducibility and allow the geometry and material properties of the orthosis to be adjusted according to patient-specific clinical and biomechanical needs [5,6,7,8,9,10].

Among these technologies, additive manufacturing has gained increasing relevance in podiatry. Fused deposition modeling and selective laser sintering enable layer-by-layer fabrication of customized insoles using polymeric materials such as PLA, TPU, or nylon. Compared with conventional techniques, additive manufacturing offers greater design freedom, controlled internal geometry, reduced material waste, and the possibility of producing lightweight and durable devices within a digital workflow [11,12,13].

The connection between plantar orthoses and biomimetics lies in the attempt to reproduce, support, or compensate for biological functions of the human foot through engineered devices. The foot is a complex biomechanical structure that combines load-bearing capacity, shock absorption, balance, and adaptation to different surfaces. Therefore, orthotic devices can be interpreted as bioinspired or bionic interfaces when their design seeks to emulate or assist these natural functions by redistributing plantar pressure, controlling local stiffness, and/or improving gait mechanics.

Additive manufacturing strengthens this biomimetic approach because it allows the fabrication of complex internal architectures that are difficult to obtain using conventional manufacturing methods. 3D-printed plantar orthoses should not be considered merely customized medical devices but engineered systems that translate biomechanical and bioinspired principles into manufacturable structures. The evaluation of recycled PLA within this framework is relevant because it addresses whether a sustainable polymer feedstock can support such biomimetic designs while maintaining the mechanical reliability required.

At the same time, the growing use of 3D printing has raised concerns about the accumulation of polymer waste generated by failed prints, support structures, prototypes, and end-of-life components. In this context, recycled polylactic acid has emerged as a promising material for circular manufacturing because of its widespread use in fused-filament fabrication and its potential compatibility with mechanical recycling strategies [14,15,16,17,18]. However, its application to plantar orthoses requires careful evaluation, since these devices must meet specific requirements related to dimensional stability, mechanical reliability, comfort, durability, and performance under repeated loading during daily use [19,20].

Recent studies have expanded the possibilities of PLA-based orthotic design. Soft PLA insoles with gyroid-like structures have shown that infill density and structural configuration influence energy absorption and compressive behavior [21]. Other studies have reported that custom PLA insoles produced by fused deposition modeling can reduce plantar pressure and improve comfort compared with conventional devices [22]. In addition, extrusion-based strategies have enabled multimaterial and functionally graded designs, including PLA/TPU systems, heating elements, shape-memory behavior, and sensor integration [23,24,25,26]. Nevertheless, most of these developments still rely primarily on virgin or engineered materials rather than recycled polymers.

Therefore, this review integrates four areas that are often addressed separately: the clinical and functional role of plantar orthoses, digital and additive manufacturing workflows, advanced extrusion-based orthotic designs, and PLA recycling strategies. The objectives are twofold: first, to assess whether recycled PLA can be processed through 3D printing for orthotic applications; and second, to define the technical and clinical conditions under which recycled-PLA foot orthoses could represent a viable and sustainable option.

### 1.1. Clinical and Technical Foundations

#### 1.1.1. Clinical Role, Typologies, and Functional Requirements of Plantar Orthoses

Plantar orthoses constitute a diverse group of devices that include prefabricated, custom-made, functional, adaptive, and hybrid designs. The type of orthotic insole prescribed depends on the therapeutic objective, which includes motion control, redistributing plantar loads, offloading vulnerable regions, structural support, improving gait mechanics, and reducing symptoms [2,3,4].

Despite their different designs and indications, all plantar orthoses share a series of common requirements. They must provide adequate support and pressure redistribution without excessive rigidity, since an overly stiff device may generate discomfort or transfer stress to adjacent areas. They should also remain compatible with footwear and daily activities, preserve hygiene and surface integrity, and maintain mechanical performance under repeated use rather than only under idealized laboratory conditions [2,3,4,11,12].

These requirements are particularly important in high-risk patients, such as individuals with diabetic foot disease, where a loss of protective sensation limits the self-regulation of loading. In such contexts, orthotic effectiveness depends not only on geometry but also on long-term fit, wearability, adherence, and durability [2,4,13].

#### 1.1.2. Digital Workflow and Applicable 3D-Printing Technologies

In clinical podiatry, additive manufacturing constitutes one of the stages within a broader digital workflow that encompasses geometry acquisition, computational design, fabrication, and clinical validation. Positioning it as a stage in insole production enables explicit control over thickness, arch support, offloading regions, and local stiffness, surpassing the constraints of purely manual fabrication [5,6,14,15,16].

A critical step in this process is the acquisition of an accurate digital model of the foot. Scan quality depends on the precision of the scanning device and the positions of both the foot and the scanner during the acquisition process. Anatomical coverage and operator consistency are also relevant to the creation of the digital model and, consequently, to the fit of the final orthosis [6,7]. For this reason, foot scanning should be considered part of a controlled clinical process, rather than a purely technical step [6,7,15].

After data acquisition, computer-aided design and subject-specific modeling allow iterative optimization of orthotic geometry and load distribution. In the modeling phase, finite element modeling (FEM) is used to analyze and predict interface pressures and to refine designs prior to manufacturing, particularly when both comfort and biomechanical performance are critically relevant [7].

Once the digital model is available, different additive manufacturing technologies may be used to fabricate the orthosis, including fused deposition modeling (FDM), stereolithography (SLA), selective laser sintering (SLS), and novel methods based on foam printing. The selection of the most appropriate technology depends on the device’s mechanical function, surface requirements, production scale, and available materials [5,14,17,18].

Recent orthotic applications support this workflow perspective. Gyroid-based FDM architectures can be designed based on patient-specific geometry and plantar-pressure distribution data, allowing the customization of damping performance and elastic energy absorption [19,20]. Similarly, customized insoles derived from scanning data and validated using plantar-pressure analysis demonstrate the value of integrating design, fabrication, and evaluation within a unified process [20].

**Figure 1:**
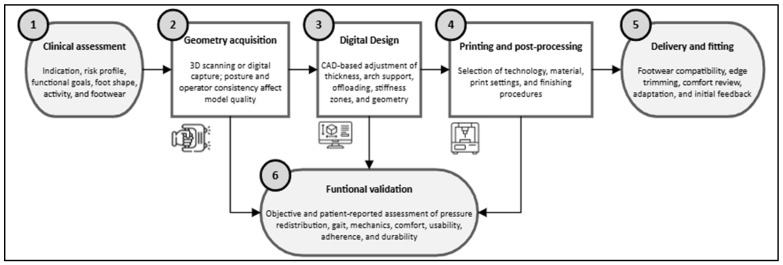
Clinical-to-digital workflow for 3D-printed plantar orthoses.

As illustrated in Figure 1, 3D printing should therefore be viewed as part of a clinical-to-digital continuum, in which final orthosis properties and performance will depend on the interaction between scanning quality, design decisions, material selection, manufacturing parameters, and validation under real-use conditions.

#### 1.1.3. Materials and Validation Criteria

A geometrically accurate orthosis may fail clinically if the material does not provide an adequate combination of stiffness, cushioning, interlayer adhesion, durability, and surface behavior under repeated use. Therefore, the use of generic printing materials for orthotic applications requires rigorous evaluation [8,14,15,21].

Several studies have explored the potential of PLA-based materials in this context. Soft PLA gyroid insoles manufactured with FDM have shown good mechanical properties, including an elastic modulus of 8.194 MPa and yield strength of 0.796 MPa, supporting their potential for energy absorption applications [19]. The use of PLA in custom soft insoles has also demonstrated suitable hardness and flexibility for integration into conventional footwear [20].

Other publications explore new directions. Multimaterial and 4D-printed systems based on PLA/TPU composites, including carbon-fiber reinforcement and carbon nanotube (CNT)-enhanced phases, demonstrate improved mechanical performance and shape-memory behavior [22]. Pellet-based manufacturing enables functionally graded structures with enhanced toughness and interfacial shear strength [17], while foam-printing approaches allow spatial control of local stiffness in orthotic geometries [18].

However, material performance cannot be assessed only through isolated mechanical parameters. Clinical validation should combine objective measures, such as plantar-pressure analysis, gait assessment, dimensional stability, fatigue behavior, and mechanical testing, with patient-reported outcomes related to comfort, usability, footwear compatibility, adherence, and frequent use. This combined approach is necessary because current evidence remains uneven: many engineering-focused studies provide detailed material characterization but limited clinical validation, whereas some clinical studies include real-use assessment but have restricted sample sizes or methodological scope [19,20,22].

## 2. Materials and Methods

This manuscript presents a critical review based on four partially overlapping lines of evidence that are relevant to studying the feasibility of recycled PLA in the 3D-printed fabrication of plantar orthoses. Scientific databases, including Scopus and Google Scholar, were used to identify relevant studies related to recycled polylactic acid (rPLA), additive manufacturing, fused deposition modeling (FDM), foot orthoses, and podiatric applications. The literature search was performed using combinations of the following keywords and search terms: “recycled PLA”, “rPLA”, “3D printing”, “fused deposition modeling”, “foot orthoses”, “podiatric devices”, “additive manufacturing”, “PLA orthotics”, “mechanical properties”, and “PLA AND foot AND orthoses”. Relevant articles were screened based on their relevance to the objectives of the present review. This approach is justified by the heterogeneity of the available studies, which range from clinical trials to materials research, and by the scarcity of direct studies on recycled PLA in orthopedic applications. This review focuses on four main domains as follows:

(i) Clinical and biomechanical evidence on foot orthoses. A key objective in the treatment of conditions such as plantar pain, diabetes, and flat feet is the redistribution of plantar pressure. Conventional orthoses have proven effective in modifying load patterns, improving alignment, and reducing the risk of ulcers. The direct influence of the orthosis’s design, stiffness, and geometry on its functional performance is corroborated by mechanical studies. Furthermore, a more precise fit and better force distribution are achieved through customization based on 3D foot scanning.
(ii) Digital workflows and additive manufacturing in podiatry and related fields. The use of 3D scanners, computer-aided design (CAD), and additive manufacturing has transformed the production of orthoses. These digital workflows enable a high degree of anatomical customization, reduced manufacturing times, rapid design iteration, and decentralized production (local manufacturing).
(iii) PLA recycling and polymer reprocessing. PLA is a bioplastic derived from renewable sources, but its main drawback is mechanical degradation after multiple processing cycles. Mechanical recycling (shredding and re-extrusion) is one method for recycling this material, but it has several drawbacks. It can lead to a reduction in molecular weight, loss of tensile and impact strength, and/or increased brittleness. However, studies show that with proper controls (temperature, humidity, number of cycles), recycled PLA can maintain sufficient properties for non-structural or semi-structural applications, such as orthoses.
(iv) Recent studies on 3D-printed orthoses made of soft or elastomeric PLA (to improve comfort and flexibility), multimaterial systems (combining stiffness and cushioning), 4D printing (materials that change shape in response to environmental stimuli), and sensorized orthoses (integration of sensors for pressure monitoring) demonstrate advances in the potential of PLA as a functional material [10,11,14,15,21,23].

The review was guided by the following question: under what technical and clinical conditions could recycled PLA become a viable material option for plantar orthoses manufactured through additive workflows?

Given the limited scientific literature that directly evaluates the use of recycled PLA in orthopedic applications, the review integrates clinical evidence on orthoses with the technical characterization of materials. The analysis addresses critical aspects such as degradation kinetics, thermomechanical reprocessing, printability, and the evolution of mechanical properties following successive recycling cycles [12,16,24,25,26].

For this reason, the extracted evidence was classified as either direct clinical evidence or indirect technical evidence. Direct clinical evidence was defined as evidence derived from orthosis-level studies involving users, plantar-pressure assessment, gait evaluation, comfort, usability, adherence, or functional outcomes. Indirect technical evidence was defined as evidence derived from material characterization, filament reprocessing, printability tests, mechanical testing of specimens, degradation studies, or process-level optimization without direct validation in orthotic use.

### 2.1. Eligibility and Source Selection

In selecting the sources, we considered those that provided relevant information to at least one of the following domains: clinical role and functional requirements of plantar orthoses; digital acquisition and CAD-based workflows; additive manufacturing technologies and materials applicable to orthotic fabrication; recycled PLA processing, degradation, and performance; and/or orthosis-relevant mechanical, functional, or clinical outcomes.

Both primary studies and review articles were considered due to the exploratory and interdisciplinary nature of the topic. Technical reports and synthesis documents were also considered when they provided structured evidence on recycled PLA behavior, processing constraints, or mechanical properties of products obtained through additive manufacturing, which also contributed relevant information [24,27].

Sources focusing exclusively on unrelated additive manufacturing applications, non-transferable devices, or purely descriptive technologies without material, functional, or clinical implications were excluded from the synthesis.

### 2.2. Data Extraction and Synthesis

To prepare this manuscript, we selected a specific set of key sources, including clinical reviews, technical guidelines on PLA recycling, and studies on 3D printing in orthopedics. It is important to note that this work is a structured synthesis of these documents and not an exhaustive literature search of databases.

Each source was chosen for its relevance, and we extracted key data such as study design, materials used, manufacturing techniques, and results (both mechanical and clinical). We then organized all of this information into four main areas: the clinical performance of the insoles, digital design and workflow, advances in 3D printing for orthotics, and the behavior of recycled PLA (degradation, printing, and improvement of its properties).

Finally, the synthesis explicitly distinguished between material-level outcomes and orthosis-level outcomes. Material-level outcomes included printability, dimensional stability, viscosity, crystallinity, interlayer adhesion, tensile or compressive properties, surface quality, and degradation after reprocessing. Orthosis-level outcomes included plantar-pressure redistribution, gait-related performance, comfort, footwear compatibility, usability, adherence, hygiene, durability under daily use, and safety. This distinction was used to avoid extrapolating material feasibility directly into clinical effectiveness.

### 2.3. Limitations of the Review Design

The main limitation identified during this review is the scarcity of literature specifically applied to clinical podiatry. There are few studies directly focused on orthotics, and most of the data on recycled PLA come from studies at the filament level or on additive manufacturing in general. Therefore, the conclusions regarding recycled PLA are mainly based on indirect technical evidence and should be interpreted as supporting potential feasibility under controlled manufacturing conditions rather than as demonstrating direct clinical effectiveness in plantar orthoses.

Accordingly, the conclusions should be interpreted as conditional and critically qualified rather than as definitive clinical validation. Although this integrative review approach is necessary given the limited body of literature in this field, it introduces a degree of methodological heterogeneity, which limits the feasibility of a formal quantitative synthesis.

## 3. Results: Design and Fabrication of 3D-Printed Plantar Orthoses

### 3.1. General Characteristics of the Included Literature

The included literature covers several partially overlapping lines of evidence. Accordingly, the analyzed studies have been organized into six groups. The first comprises clinical and biomechanical reviews addressing foot orthoses, diabetic foot offloading, footwear comfort, and orthotic efficacy [1,2,3,4,11,25]. The second focuses on digital workflows, including foot scanning, user-specific CAD, CAM, and CAE, and 3D-printed orthoses [5,6,7,8,9,10,14,15]. The third addresses PLA reuse and recycling, primarily through technical reports and reviews derived from general additive manufacturing rather than specific research on orthoses [12,13,16,21,23,26,27,28,29]. The fourth consists of studies using soft PLA for flexible components in insoles [19,20,22], while the fifth explores orthopedic insoles based on multimaterial architectures [17,18,19,20,22,30]. Finally, the sixth group focuses on cost reduction, parametric design, and workflows using recycled materials [13,16,22,31]. These groups were further interpreted according to the level of evidence they provide for recycled PLA in plantar orthoses. Clinical and orthosis-focused studies were considered direct or near-direct evidence when they assessed device-level function, whereas recycled-PLA studies based on filaments, specimens, or processing conditions were considered indirect technical evidence.

In all the aforementioned lines of research, the priority is placed on design, production, and initial performance rather than on using recycled materials. Consequently, the available scientific literature is skewed. Studies focused on orthoses provide information and results on the use of soft PLA, gyroid structures, customized geometries, and multimaterial solutions. Meanwhile, recycled-PLA research remains dominated by indirect evidence at the filament or specimen level [24,27,32].

This asymmetry highlights that progress in orthotic design and manufacturing has outpaced the direct validation of recycled feedstocks for podiatric use. Recycled PLA can therefore be considered a technically relevant candidate material, but not an orthosis material with the same degree of direct clinical validation as materials already evaluated in device-level studies.

**Figure 2:**
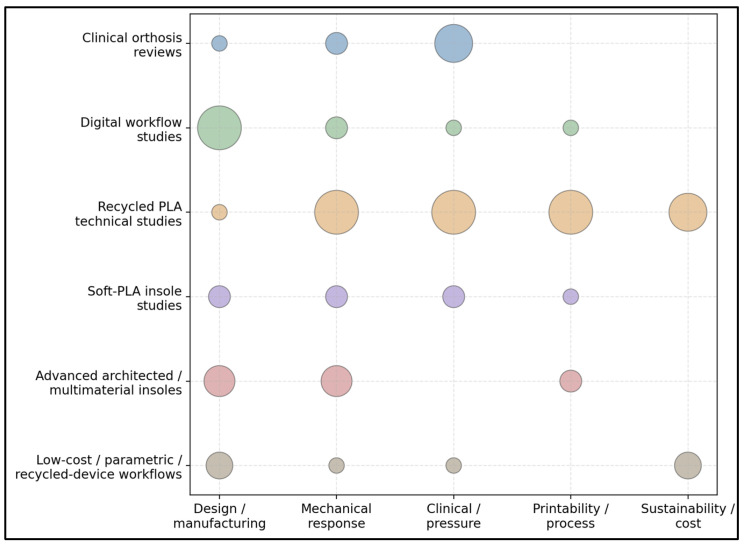
Evidence map of the reviewed literature on 3D-printed plantar orthoses and recycled PLA.

Figure 2 summarizes the distribution of the reviewed evidence according to type and outcome domain. The map shows that orthosis-specific studies are concentrated mainly on design, manufacturing, and preliminary functional assessment, whereas recycled-PLA studies are concentrated on material behavior, printability, and process-related outcomes. Overall, the field is advancing more rapidly in the engineering of customized insoles than in the direct orthosis-level validation of recycled feedstocks.

### 3.2. Design Trends and Manufacturing Parameters

The literature indicates that orthotic performance depends on the interaction between geometry, thickness distribution, internal architecture, and manufacturing parameters. Additive manufacturing enables explicit control over infill density, shell count, local reinforcement, and offloading features, offering greater design flexibility than conventional workflows [5,7,8,16,21].

Representative extrusion settings reported for recycled materials include flat-bed build orientation, 0.2 mm layer height, two shell layers, 100% infill, and ±45° infill orientation in benchmark specimens [27]. While these parameters are not universal orthotic standards, they illustrate the strong influence of process definition on mechanical part behavior.

In the case of recycled PLA, it is particularly important to adapt the additive manufacturing process. Various technical reports indicate that fully recycled PLA may require higher nozzle temperatures than virgin PLA. Several publications on the viability of recycled PLA have reported that a high printing temperature is necessary to obtain the best mechanical conditions; for instance, nozzle temperatures for recycled PLA are higher than those for virgin PLA, which typically range from 190 to 210 °C [23,28]. These adjustments to the additive manufacturing process have direct implications for process control and reproducibility.

Alongside many technical trials on recycled materials, experimental studies have also been conducted with patients, providing direct clinical evidence. Recent orthosis-focused studies reinforce the importance of design variables. Jonnala et al. used a soft PLA gyroid architecture to demonstrate that lattice topology and infill density are key determinants of compressive response and energy absorption [19]. Their design was fabricated using 0.15 mm layers, 220 °C nozzle temperature, and 100% infill, emphasizing CAD-based customization to maximize additive manufacturing capabilities in insole production [19]. Similarly, Ravi Kumar et al. developed a workflow in which scanned foot geometry was converted into a 3D-printed custom soft PLA insole, followed by cleaning and finishing before plantar-pressure testing [20]. Although structurally less complex than the gyroid configurations, this study highlights the balance between process parameters and material softness in balancing manufacturability and comfort.

Low-cost and parametric workflows suggest an increasing automation of orthotics. Design-oriented studies report reduced infill values of approximately 14% in cushioning-focused concepts, using parametric tools such as Grasshopper to adapt orthotic geometry to anatomy and baropodometric data, including local densification and support adjustments [31,33,34,35].

Advanced studies indicate a broader trend toward architected, graded, and multifunctional insoles. Zhang et al. fabricated region-specific support-unit-cell structures, whereas Goh et al. used pellet-based multimaterial printing to create graded PLA/TPU transitions in a multifunctional insole combining support, cushioning, and heating elements [17,22]. Emery et al. extended local mechanical tuning to foam-based orthotic geometries by demonstrating spatially programmed stiffness zones [18]. Collectively, these studies suggest that current design trends are moving beyond simple shape customization toward the spatial programming of mechanical response.

Table 1 compares the main materials analyzed for the additive manufacturing of foot orthoses, including virgin PLA, recycled PLA, soft PLA, TPU, and multimaterial or degradable systems based on PLA/TPU. This comparison indicates that material selection cannot be evaluated solely on printability. On the contrary, an optimal solution requires a balance between structural support, damping behavior, interlayer integrity, durability, and process stability. For recycled systems, this evaluation must strictly incorporate feedstock traceability and property uniformity throughout the reprocessing cycles.

### 3.3. Functional and Clinical Evidence

The reviewed clinical and biomechanical literature supports the view that 3D-printed plantar orthoses can achieve functionally relevant pressure redistribution and biomechanical effects when design and fabrication are appropriately matched to the intended use [4,8]. Comparative studies also suggest that 3D-printed orthoses may approach the mechanical and biomechanical behavior of conventionally manufactured devices, although the available evidence remains limited and methodologically heterogeneous [15].

It is important to highlight that these clinical and functional data should not be interpreted as direct validation of recycled PLA. Most studies on orthoses evaluate virgin PLA, soft PLA, TPU, or specifically designed multimaterial systems. Therefore, while their results support the feasibility of additive manufacturing for foot orthoses, they provide only indirect information regarding the potential use of recycled PLA.

Among the recent primary studies, Ravi Kumar et al. reported that a custom soft PLA insole reduced pressure in the forefoot and hindfoot during static testing by approximately 30% and 17%, respectively. During dynamic testing, pressure reductions were observed at the hallux, metatarsal I, metatarsals II–V, the lateral arch, and the heel, whereas pressure increased in the lateral toes and medial midfoot. The authors interpreted the overall pattern as compatible with improved gait comfort and more favorable foot-loading characteristics [20].

In contrast, the study by Jonnala et al. on gyroid patterns focused more on structural design and the characterization of compressive strength than on clinical validation [19]. The authors performed custom fabrication for each patient but did not evaluate clinical efficacy, long-term comfort, durability, or wear resistance [19]. This highlights a recurring pattern in research in the field: some studies are highly developed in terms of design and materials, while others provide preliminary functional or clinical data but with much more limited technical characterization.

More exploratory studies on insole development have helped expand the functional scope of the literature. Notable in this regard is the work by Goh et al., who proposed a multimaterial insole for diabetic feet, consisting of three layers: a rigid base composed mainly of PLA, a cushioned layer, and a conductive heating layer, both TPU-based [17]. Saini and Mandal developed a concept for a smart insole made of PLA capable of monitoring foot pressure distribution and recovering energy during walking [30]. Although these studies expand the potential applications of orthoses developed using extrusion techniques, they are proof-of-concept designs.

### 3.4. Post-Processing, Finishing, and Long-Term Use Behavior

In the reviewed literature, information on post-processing was inconsistent, despite its relevance. In studies on custom soft PLA insoles, it was found that the printed parts required mechanical cleaning to improve the surface finish due to the presence of surface irregularities [20,56,57,58]. Several studies also indicated the need for post-processing to improve surface quality in designs with gyroid patterns [19,26,39,59,60,61]. These findings are particularly relevant, as foot orthoses are not isolated structural prototypes but come into direct contact with the user.

Research findings on long-term performance are limited. The existing literature provides little information on fatigue resistance, wear, or long-term stability [17,18,19,20,22,30]. This limitation is particularly relevant in the case of recycled PLA, as there is a high degree of uncertainty regarding the durability of the orthoses.

Overall, the reviewed literature indicates that long-term performance is an area with limited evidence. Existing studies support the feasibility of manufacturing custom orthoses via extrusion and suggest promising short-term functional outcomes, but there is little evidence regarding long-term use.

## 4. Results: Reuse and Recycling of PLA for Podiatric Applications

Based on a review of the technical literature, processing recycled PLA remains highly material-dependent rather than serving as a sustainable substitute. In podiatric applications, not only is it relevant that the PLA can be recycled, but parameters like dimensional consistency, interlayer integrity, fatigue resistance, surface quality, and functionally relevant performance in the final manufactured orthosis are also critical [37,38,39,40,62,63,64,65,66,67,68].

### 4.1. Operational Definitions and Recommended Processing Ranges

Table 2 summarizes the main variables in the process and indicates the ranges reported in the literature for recycled PLA in FDM-oriented podiatric applications [11,16,19,20,69,70,71,72,73,74,75,76].

It is important to control these variables because they alter the stiffness, damping, and fatigue response of the final device, which is subjected to gait-related loading. Comparative reviews indicate that recycled-PLA feedstock can be processed for orthotic fabrication, although usually under more limiting process windows than virgin feedstock. These limitations are highlighted by reported reductions of approximately 15% in tensile strength and around 17% in intrinsic viscosity after repeated reprocessing [24,39,56,57,58,64]. In addition, strict moisture control is necessary, with humidity levels below approximately 0.02% recommended to reduce hydrolytic degradation during filament processing and printing [39].

### 4.2. Degradation Mechanisms During Recycling

Table 3 summarizes the different degradation pathways found in the literature, which promote changes in molecular weight, changes in melt viscosity that increase crystallinity, and cumulative oxidative damage. All of them may affect print stability and the long-term mechanical reliability of the final orthotic device [38,40,48,62,63,64,65].

#### 4.2.1. Viscosity and Thermal Degradation

In the extrusion and printing processes, thermal degradation occurs via random polymer-chain scission. As a result, the melt viscosity is reduced, and the melt flow index increases. These changes compromise the filament’s dimensional stability, interlayer adhesion, and structural support during FDM printing [36,39]. However, modification of the viscosity of rPLA systems can be accomplished through composition adjustments and the incorporation of chain compatibilizers. Increasing the PLA concentration fundamentally raises the solution viscosity, e.g., from 41.87 to 339.83 mPa·s, promoting crucial changes that transition the morphology from beaded to stable, larger-diameter bead-free fibers [77]. Furthermore, multifunctional additives (e.g., high epoxy-functionality polymers like HSMG) may significantly increase the melt viscosity and lower the melt flow index, resulting in a refined dispersed phase morphology and mechanical properties comparable to those of general high-impact resins [78]. In terms of podiatry, all these changes are relevant to manufacturing stability since these kinds of devices depend on repeatable geometry and reliable wall formation.

#### 4.2.2. Changes in Crystallinity and Molecular Weight

The semicrystalline structure of PLA is altered by mechanical reprocessing because shorter polymer chains are more movable. Despite reduced molecular weight, recycled PLA may exhibit increased crystallinity. Munoz-Shuguli et al. [79] found that PLA crystallinity increased from 6.9 to 39.5% after six reprocessing cycles, while the molecular weight was reduced by up to 40%. A moderate increase in crystallinity can increase stiffness. However, excessive crystallinity may reduce impact resistance and make the material less suitable for devices exposed to repeated gait-related loads [40,63].

#### 4.2.3. Oxidative Degradation

During recycling, exposure to oxygen and moisture causes oxidative reactions and the formation of carbonyl groups, while also reducing the molecular weight. These effects can accumulate over successive cycles and may contribute to progressive loss of mechanical performance as well as the visible yellowing of printed parts [64].

### 4.3. Characterization of Recycled-PLA Filament and Printed Parts

Recycled PLA should be characterized at both the feedstock and printed-part levels because the clinically relevant unit is the finished orthosis. Variables include dimensional tolerances, mechanical strength, surface roughness, hardness, and fatigue life—variables that are relevant to foot biomechanics applications [23,62,65,66,67]. However, most available recycled-PLA studies stop at the feedstock, filament, specimen, or printed-part level and do not evaluate complete orthoses under clinical conditions.

Table 4 summarizes the indicative values reported for virgin and recycled PLA-printed parts in the review. These values are obtained from heterogeneous studies, and they show the proportions of expected property changes [40,65,66,67].

In the reviewed literature, a greater filament diameter variation was found in recycled PLA, approximately 0.07 mm compared with around 0.03 mm in virgin filament, which may affect extrusion consistency and final orthosis fit [38,40,65]. The reduction in mechanical resistance is associated with repeated recycling, while surface roughness tends to increase and hardness may rise slightly as a consequence of increased crystallinity [40,66]. Fatigue behavior appears particularly relevant for orthotic components exposed to cyclic gait loading [67].

### 4.4. Strategies to Mitigate Degradation

Several strategies for mitigating degradation were identified in the reviewed studies. One of the most common strategies is to blend recycled PLA with 30–50% virgin PLA, as this combination can partially offset the degradation suffered by the recycled material while retaining some of its sustainability benefits [29]. Some studies propose chain extenders and reactive additives, including systems based on epoxy and dianhydrides, to reconnect polymer chains, increase melt viscosity, and improve crack propagation resistance, although their use requires attention to safety and biocompatibility [65,81]. Controlling humidity during storage and processing is repeatedly highlighted as a critical measure, given that hydrolytic degradation can rapidly compromise the quality of the raw material [29,37].

Mitigation strategies are not limited solely to the molecular level; they can also be addressed by redesigning the manufacturing process itself. In this regard, reducing residence time and shear exposure, increasing the number of perimeters, optimizing layer height, and employing reticular or graded architectures can help compensate for some of the structural loss associated with recycling [39,62]. Likewise, solid-state polycondensation has been proposed as a post-recycling treatment capable of partially restoring molecular weight without the need to add external agents [65].

### 4.5. Selected Technical and Application-Oriented Findings

Comparative studies indicate that fully recycled PLA may require higher nozzle temperatures than virgin PLA to maintain stable extrusion, while also showing reductions in tensile strength of approximately 15% under comparative testing [24]. Also, source-controlled recycled PLA from known waste may be used for up to three extrusion cycles without deterioration under selected conditions [64].

From a material perspective, recycled PLA performance cannot be rated only in terms of printability, because mechanical recycling has also been associated with increased crystallinity and reductions in intrinsic viscosity of around 17%. These changes may affect stiffness, process stability, and brittleness [39]. Concerning orthosis design studies, soft PLA gyroid structures have shown an elastic modulus of 8.194 MPa and an elastic energy absorption of 0.0458 J/mm^3^ in insole-oriented specimens [19]. Moreover, low-cost customized insole workflows based on TPU, PLA, and ABS have reported direct fabrication costs of approximately €7.30 per device, with low infill levels of around 14% explored in cushioning-oriented designs [31].

Beyond PLA-specific studies, more extensive recycled-polymer workflows have demonstrated that waste-derived materials can be converted into functional personalized assistive devices, including orthotic and prosthetic applications [33]. In parallel, parametric design environments have shown that baropodometric data can be incorporated into automated geometric adaptation and local densification strategies for targeted offloading [34]. All of these studies indicate that recycled PLA should be considered within a broader technological context that includes thermal control, feedstock traceability, structural design, and patient-specific digital workflows. Nevertheless, these findings remain indirect with respect to clinical orthotic performance unless they are confirmed in complete plantar orthoses tested with users.

### 4.6. Mechanical Recycling Workflow of PLA for Podiatric Devices

Mechanical recycling in podiatric manufacturing is relevant because it enables decentralized production, which allows for the recovery of material from failed prints and expired components, reducing raw-material costs. This mechanical recycling chain found in the reviewed technical sources contains collection and sorting, washing, shredding, drying, extrusion, and either filament production for FDM or direct fused granulate fabrication (FGF) from pellets or flakes [37,39,40,62,63].

**Figure 3:**
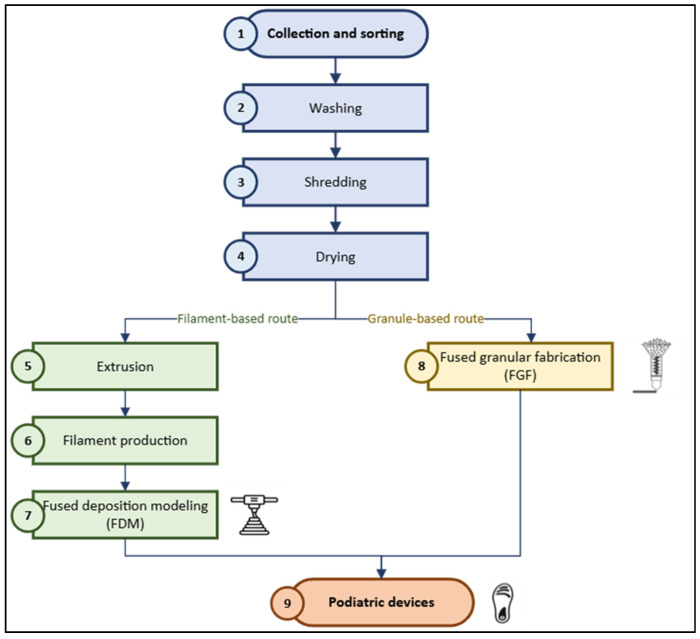
Mechanical recycling workflow of PLA for podiatric devices.

Figure 3 illustrates the mechanical recycling workflow of PLA for podiatric manufacturing. The first step in this process is collecting and sorting the material, followed by washing, shredding, and drying as pre-treatment. After drying, the recycled material can follow two paths: it can either be re-extruded into filament for FDM or processed directly as granules through FGF. This difference between the two paths is decisive because the first one introduces additional transformation stages, whereas the second one may reduce intermediate processing before device fabrication. In both cases, the final objective is the production of podiatric devices.

Also, the background research indicates that each reprocessing cycle introduces thermal and mechanical stresses that promote chain scission and oxidation. Without mitigation strategies, this progressive degradation has a propensity to reduce tensile strength and elongation at break and may limit reuse to two to four cycles, depending on feedstock history and process control [28,38,39,62,68].

## 5. Results: Cost, Feasibility, and Sustainability

### 5.1. Technical Feasibility

The reviewed literature indicates that the technical feasibility of using recycled PLA in the manufacture of podiatric products is limited. Recycled PLA is not described as a substitute for virgin raw material, and its feasibility depends on several factors, including the origin of the raw material, the number of recycling cycles, the quality of the filament, the adaptation of thermal processing conditions, and mechanical requirements [24,26,27,32].

Available studies indicate that feasibility may vary depending on the type of application. Thus, the use of recycled PLA appears more suitable for the development of prototypes, design iterations, educational models, or components with lower mechanical demands than for orthoses intended to withstand high and repetitive plantar loads during long-term daily use [5,15,23,26].

Recent studies focused on orthoses also indicate that high-performance devices rely on controlled architectures, local stiffness gradients, and integrated functionalities [21,22,23,24,25,26]. This trend imposes stricter requirements on material consistency and manufacturing process stability, especially in advanced orthotic applications.

### 5.2. Economic and Environmental Feasibility

The reviewed technical reports consistently describe an economic rationale for PLA recycling, mainly because reprocessing waste into reusable feedstock may reduce material costs compared with the use of virgin pellets or filament [24,26,32]. They also describe an environmental rationale based on lower dependence on virgin raw materials and reduced lifecycle burdens relative to linear models of manufacturing and disposal.

Within the orthotic field, this perspective is consistent with broader sustainability-oriented work in device design and fabrication. Case studies in orthotic and footwear-related applications have emphasized the relevance of material selection, manufacturing route, durability, and lifecycle thinking during device development [11,25].

Some low-cost customized insole workflows have reported direct fabrication costs of approximately €7.30 per device, while recycled-plastic case studies have described reductions in production costs relative to conventional routes, particularly in low-resource settings [31,33]. However, these figures derive from heterogeneous workflows and should be interpreted cautiously because they depend on equipment, labor, workflow maturity, and comparator conditions.

The reviewed literature also highlights that greater technical complexity can lead to new challenges. The use of multiple materials, conductive layers, and temperature-sensitive elements can enhance functionality, but these additions can also increase manufacturing complexity and reduce recyclability or repairability [17,22,30].

### 5.3. Limits to Transfer into Podiatric Practice

The evaluated data indicate that economic and environmental advantages alone are insufficient to establish clinical appropriateness. Most of the available recycled-PLA evidence derives from generic additive manufacturing studies, filament reprocessing studies, or standardized mechanical specimens, rather than from direct plantar-orthosis trials involving human participants [24,26,27,66,67,68].

Consequently, the extrapolation of results obtained from recycled filaments or standardized samples to the functional performance of an orthosis is indirect. Such extrapolation should therefore be treated as hypothesis-generating rather than confirmatory. The available information regarding orthoses made from recycled PLA in relation to clinical and real-world variables, such as plantar-pressure redistribution, comfort, hygiene, fatigue resistance, and safety, is limited [8,11,13].

## 6. Discussion

Taken together, the available reports suggest that manufacturing technology, design decisions, material properties, and clinical performance form an interdependent system and should not be evaluated in isolation. Additive manufacturing is particularly attractive in podiatry due to its ability to combine customization, reproducibility, and digital process control; however, this high degree of precision means that any material inconsistency is more easily translated into variations in device behavior and final performance [5,6,7,8,9,23].

In this context, virgin PLA and recycled PLA should not be considered equivalent materials. They differ not only in their environmental profile but also in their molecular structure, mechanical response, and rheological behavior [57]. Virgin PLA is generally characterized by a more controlled molecular weight distribution, more predictable chain architecture, and a more stable thermal and processing history [65]. In contrast, recycled PLA has already undergone one or more thermomechanical processing stages, which may induce polymer-chain scission, hydrolytic degradation, and oxidative reactions. These mechanisms tend to reduce molecular weight and may generate shorter and more mobile polymer chains.

At the molecular level, the reduction in molecular weight resulting from a slight thermal degradation is one of the main consequences of PLA recycling. Shorter chains may increase chain mobility and favor changes in crystallinity [82]. As a result, recycled PLA may become locally stiffer due to increased crystallinity but also more brittle and less tolerant to impact or cyclic loading. This is particularly relevant for plantar orthoses, because these devices are subjected to repeated gait-related loads rather than isolated static loading conditions.

From a mechanical perspective, recycled PLA generally shows lower tensile strength, reduced toughness (5% to 15% reduction), lower elongation at break, and more limited fatigue resistance than virgin PLA [65]. Although some stiffness may be preserved, or even increased in some cases due to crystallinity changes, this does not necessarily imply improved orthotic performance [83]. Excessive brittleness, reduced crack propagation resistance, and poorer fatigue behavior may compromise durability, especially in regions of the orthosis exposed to repeated bending, compression, or local stress concentrations.

The rheological behavior of recycled PLA is also different from that of virgin PLA. Mechanical recycling and repeated extrusion may reduce melt viscosity and increase the melt flow index, affecting filament diameter stability, extrusion regularity, and layer deposition during fused-filament fabrication [83,84]. These changes can narrow the processing window and may require adjustments in printing parameters, such as nozzle temperature, printing speed, cooling conditions, or layer height. In FDM-based plantar orthoses, rheological instability is particularly important because it may affect dimensional accuracy, interlayer adhesion, surface finish, and local mechanical consistency.

Therefore, recycled PLA should not be treated as a direct substitute for virgin PLA. While virgin PLA provides a more predictable benchmark material for additive manufacturing, recycled PLA requires additional control of feedstock origin, moisture content, recycling history, filament quality, and printed-part properties. For orthotic applications, the relevant question is not only whether recycled PLA can be printed but also whether the final printed orthosis can maintain adequate mechanical reliability, dimensional stability, comfort, and durability during use.

The recycling process and the number of recycling cycles further intensify these differences. Mechanical recycling usually involves collection, sorting, washing, shredding, drying, extrusion, and either filament production for FDM or direct granule-based fabrication. Each of these stages may introduce additional thermal, mechanical, and moisture-related stresses. As the number of recycling cycles increases, cumulative chain scission, oxidation, and hydrolytic degradation may progressively reduce molecular weight, modify melt viscosity, increase brittleness, and reduce tensile strength, elongation at break, and fatigue resistance. Therefore, the recycling history of PLA is not a secondary variable but a key determinant of material reliability. A recycled-PLA feedstock subjected to one controlled reprocessing cycle cannot be assumed to behave in the same way as material that has undergone several uncontrolled cycles. For plantar orthoses, this is particularly relevant because small variations in filament quality, viscosity, crystallinity, and interlayer adhesion may affect not only printability but also long-term performance under repeated gait-related loading.

Mixing virgin and recycled PLA represents a practical strategy to balance sustainability and functional reliability. The addition of virgin PLA can partially compensate for the loss of molecular weight and mechanical performance associated with recycled PLA, while also improving process stability, filament regularity, and reproducibility during extrusion-based printing. From an environmental perspective, these blends still reduce the consumption of virgin polymer, although to a lesser extent than fully recycled feedstocks. From a clinical and technical perspective, however, blended materials may be more realistic for near-term orthotic applications because they reduce the risks associated with fully recycled PLA. The optimal proportion of recycled PLA should depend on the functional demand of the orthotic component. Higher recycled contents may be suitable for prototypes, fitting trials, educational models, or low-load regions, whereas definitive orthoses, load-bearing regions, or devices intended for long-term use should rely on more conservative blends and require validation of printed-part properties and orthosis-level performance [24].

The increasing use of glass- and carbon-fiber-reinforced filaments also has important implications for recyclability and further use. Fiber reinforcement can improve stiffness, dimensional stability, and load-bearing capacity [85], which may be attractive for selected structural zones of plantar orthoses. However, reinforced PLA should not be treated as equivalent to unfilled PLA from a circular-economy perspective. The presence of glass or carbon fibers creates a composite waste stream that is more difficult to recycle mechanically, because shredding, extrusion, filtration, and reprocessing must preserve not only the polymer matrix but also the fiber length, dispersion, orientation, and matrix–fiber adhesion. Repeated recycling may shorten or damage the fibers, reduce reinforcement efficiency, increase heterogeneity, and lead to more abrasive feedstock for FDM nozzles. Consequently, although fiber-reinforced PLA may improve mechanical performance in the first use cycle, it can reduce material circularity and complicate further reuse unless a specific end-of-life handling and reprocessing route is defined.

The viability of recycled PLA depends on the context of use and application requirements rather than serving as a universal solution. The reviewed literature indicates that poorly controlled feedstock, multiple recycling cycles, and filament inconsistency compromise orthosis reliability, even when printability is preserved. Conversely, when recycling history is limited, process parameters are adapted, and device function is matched to material capability, recycled PLA represents a viable component within a more sustainable additive workflow [7,47,52].

Recent studies further refine this interpretation. On one hand, extrusion-based technologies can produce orthoses with sophisticated structural logic, including architected infill geometries for energy absorption, soft PLA components evaluated under static and dynamic pressure conditions, and multimaterial systems combining support, cushioning, and additional functionalities such as heating [17,26,86]. On the other hand, these studies highlight the absence of a consolidated validation framework. Clinical effectiveness, long-term durability, user comfort, and hygiene are assessed inconsistently, particularly in more advanced or sensorized designs.

This imbalance has direct implications for recycled PLA. While the orthosis field is progressively defining performance benchmarks, the recycled-PLA literature has not yet demonstrated that such benchmarks can be consistently achieved. As a result, the evidentiary threshold for recycled materials becomes increasingly demanding as orthotic design evolves.

Consequently, the evidence supports a graded implementation strategy rather than universal adoption. Recycled PLA is more readily applicable in prototyping, educational contexts, non-critical components, or controlled, lower-demand applications. Its use in definitive plantar orthoses exposed to sustained, repetitive loading remains promising but insufficiently validated [5,15].

Finally, emerging developments such as sensorized insoles, spatially programmed materials, 4D-printed structures, and multimaterial graded systems provide a useful reference framework for future orthotic design. Their relevance in this review lies not in immediate clinical transfer, but in illustrating the increasing level of control over local mechanical behavior, comfort, and function achievable through additive manufacturing. Within this evolving landscape, recycled PLA should be evaluated not only in terms of printability but also in relation to its capacity to meet clinically relevant performance standards.

### 6.1. Direct and Indirect Evidence Supporting Recycled PLA

A key finding of this review is that the evidence supporting the use of recycled PLA for foot orthoses is mixed. On the one hand, direct clinical evidence is available for some designs of 3D-printed orthoses, including studies evaluating plantar-pressure redistribution, comfort, or functional performance. However, these studies typically use virgin PLA, soft PLA, TPU, or engineered multimaterial systems, rather than recycled PLA. In contrast, the literature on recycled PLA provides technical validation regarding printability, degradation, viscosity, crystallinity, dimensional consistency, and mechanical behavior following reprocessing but rarely evaluates complete foot orthoses under real or simulated clinical conditions.

Therefore, the current validation of recycled PLA in foot orthoses should be interpreted as indirect technical evidence of feasibility, not as direct clinical validation. This distinction is essential because it cannot be assumed that performance at the material level automatically translates into safety, comfort, durability, or therapeutic efficacy at the orthosis level.

### 6.2. Research Gaps and Recommendations

Integrated analysis of the clinical and technical literature reveals recurrent gaps regarding the use of recycled PLA in plantar orthoses.

A first gap relates to the lack of standardization. Reviewed studies differ substantially in feedstock origin, recycling workflows, build orientation, processing temperatures, and outcome measures, which limits comparability and hinders translation into device-level recommendations [6,15,26,29,50,52,59].

A second gap concerns traceability. Clinically meaningful use of recycled materials requires detailed documentation of feedstock origin, recycling cycles, additives or contamination, and processing conditions. Without such traceability, reproducibility and regulatory evaluation are compromised [6,9,10,15,72].

A third gap is the limited availability of real-use validation. Current evidence provides insufficient data linking recycled-material behavior to plantar-pressure redistribution, gait-related outcomes, comfort, hygiene, footwear interaction, and long-term adherence. Most studies address either material properties or early functional outcomes, but they rarely address both within the same design framework [12].

A fourth gap reflects an imbalance between design innovation and clinical validation. While advanced orthotic concepts, including gyroid lattices, multimaterial gradients, 4D-printed structures, and sensorized or thermally active insoles, are developing rapidly, evidence on durability, maintenance, user experience, and comparative clinical effectiveness remains limited [19,20].

Together, these gaps suggest that future research should prioritize several directions. First, standardized reporting of recycled feedstocks and processing parameters is needed to improve comparability across studies. Second, integrated testing frameworks combining mechanical characterization and clinical evaluation of the final orthosis should be developed. Third, direct comparisons between virgin and recycled materials under equivalent orthotic designs are required. Fourth, lifecycle assessments should incorporate both environmental impact and long-term durability. Finally, study designs should compare simplified single-material orthoses with complex, multifunctional systems under clinically relevant conditions [11,25].

## 7. Conclusions

Foot orthotics are essential in podiatric practice to alter load distribution, improve biomechanical function, and provide preventive and therapeutic value across various clinical conditions (e.g., diabetic foot) [70,71,87]. Additive manufacturing has expanded customization capabilities by integrating digital geometry acquisition, computational design, and controlled manufacturing processes within a single workflow [7]. This integration improves device geometry and enables the development of complex geometries, differentiated functional zones, and tailored load-relief strategies.

Recent literature confirms that extrusion technologies support custom insoles, engineered structures, multimaterial configurations, and emerging functional concepts [2,7,13,14,22,69,70]. These advancements overcome the limitations of traditional manufacturing by offering new pathways to modulate support, cushioning, local stiffness, and comfort. However, technological development does not imply functional or clinical equivalence among all feedstock materials. In particular, incorporating recycled polymers poses additional challenges regarding consistency, traceability, processing stability, and final performance validation.

Recycled PLA is a promising alternative for sustainability, waste reduction, and circular production streams [58,61,67]. However, recycling processes can significantly alter material behavior. Key degradation mechanisms include reduced molecular weight, alterations in viscosity, increased crystallinity, cumulative oxidative degradation, and dimensional variability in the extruded filament. These effects can be partially offset through thermal control, drying processes, blending with virgin material, additives, or redesign of the process and the part’s architecture. However, their presence introduces some variability in mechanical behavior under actual clinical use conditions.

Therefore, the main conclusion of this review is that recycled PLA should not currently be considered a direct, universal substitute for virgin PLA in foot orthoses. Its viability remains conditional upon feedstock origin and traceability, the number of reprocessing cycles, filament quality, printing parameters, part architecture, and, above all, the specific biomechanical function required.

## Figures and Tables

**Table 1:** Material comparison for 3D-printed orthotic use.

Material/ System [References]	Support/Stiffness	Cushioning	Printability	Recyclability	Orthotic Suitability	Main Caveat	Evidence Level
**Virgin PLA [16,19,20,24,26,27,32]**	High	Moderate–low	High	Moderate	Useful benchmark for rigid or structurally stable printed parts	Relatively brittle in cushioning-oriented designs	Direct/near-direct orthosis evidence and technical evidence
**Recycled PLA (rPLA) [26,29,36,37,38,39,40]**	Moderate and variable	Moderate	Moderate to low	High	Conditionally viable where traceability and process control are strong	Degradation, feedstock variability, narrower process window	Mainly indirect technical evidence
**Soft PLA [19,20,41,42,43,44]**	Moderate	High	High	Unclear/not established	Promising for comfort-oriented or cushioning insoles	Limited long-term validation and no direct PLA translation	Direct/near-direct orthosis evidence, but not recycled-PLA evidence
**TPU** **[17,22,45,46,47,48]**	Moderate	High	Moderate	Moderate	Useful for flexible or compliant orthotic regions	Lower structural support than stiffer PLA-based systems	Orthosis-related evidence, mainly technical and functional
**PLA/TPU blends or graded** **interfaces [17,22,45,46,47,48]**	Tunable	High	Moderate	Low to moderate	Promising for zonal support plus cushioning	Manufacturing complexity and little clinical validation	Exploratory technical/proof-of-concept evidence
**Reinforced/** **architected PLA systems** **[18,22,49,50,51,52,53,54,55]**	High to very high	Tunable	Moderate	Low	Experimental route for spatially programmed support	Exploratory and not recycled-PLA validated	Exploratory technical evidence

**Table 2:** Operational variables and indicative processing ranges for recycled PLA in podiatric FDM applications.

Variable	Operational Definition	Typical Range for rPLA	Orthotic Relevance
**Extrusion temperature**	Temperature at which PLA is melted and homogenized before filament formation	170–190 °C	Excessive heat accelerates degradation before printing
**Nozzle temperature**	Printing temperature during FDM	195–215 °C	Affects layer adhesion and surface finish
**Printing speed**	Linear velocity of nozzle movement	30–60 mm/s	Higher speeds increase dimensional deviation
**Melt Flow Index (MFI)**	Indicator of melt viscosity	Typically increased vs. virgin PLA	Influences filament diameter stability and flow consistency
**Cooling rate**	Solidification rate after deposition	Moderate, e.g., fan 50–70%	Controls crystallinity, warpage, and interlayer consolidation

**Table 3:** Main degradation effects reported for mechanically recycled PLA and their orthotic implications.

Property	Effect of Recycling	Potential Impact on Podiatric Use
**Molecular weight [39,40]**	Decreases	Reduced toughness and greater brittleness
**Viscosity [38,64]**	Often decreases overall	Print instability and poorer deposition control
**Crystallinity [39,63]**	Tends to increase	Higher stiffness but lower impact tolerance
**Oxidation [40]**	Accumulates over cycles	Long-term mechanical decay and yellowing

**Table 4:** Indicative property ranges for virgin and recycled-PLA-printed parts reported in the uploaded technical section [40,65,67,80].

Property	Virgin PLA	Recycled PLA (2–3 Cycles)
**Tensile strength (MPa)**	55–65	40–50
**Surface roughness Ra (µm)**	Lower	Higher
**Shore D hardness**	Similar or slightly higher	Similar/slightly increased
**Fatigue resistance**	Higher	Moderate

## Data Availability

No new data were created in this study. Data sharing is not applicable to this article, as the manuscript is a review based on previously published literature. The sources analyzed are listed in the References Section.
